# Cost of reusable vs. single-use ureteroscopes in complex renal surgeries: a randomized cohort study

**DOI:** 10.1007/s00345-025-05957-y

**Published:** 2025-11-10

**Authors:** Rei Unno, Kazumi Taguchi, Shuzo Hamamoto, Takahiro Yanase, Kengo Kawase, Teruaki Sugino, Takahiro Yasui

**Affiliations:** 1 Department of Nephro-urology, Nagoya City University Graduate School of Medical Sciences, 1 Kawasumi, Mizuho-cho, Mizuho-ku, Nagoya, 467-8601 Japan; 2 Department of Urology, University of Alabama at Birmingham, Birmingham, United States

**Keywords:** Endoscopic combined intrarenal surgery, Cost-effectiveness, Ureteroscope, Single-use, Reusable scope

## Abstract

**Purpose:**

To conduct a cost-benefit analysis comparing the latest generation digital reusable ureteroscope (URS) with single-use scopes in endoscopic combined intrarenal surgery (ECIRS).

**Methods:**

A single-center, randomized cohort study was conducted during September 2021–June 2024, comparing the two types of URS. Patients undergoing ECIRS were randomized into either the URF-V3 or 7.5Fr flexible single-use URS group based on their physical and stone characteristics. Five newly acquired URF-V3 scopes were utilized. Percutaneous lithotomy was performed using a laser and LithoClast, whereas laser lithotripsy was conducted via URS. The primary endpoints included the cost per procedure and scope durability, whereas the secondary endpoints focused on surgical outcomes.

**Results:**

Overall, 178 patients undergoing ECIRS (*n* = 89 per group) were analyzed. Four single-use scopes broke, and the URF-V3 scopes required six repairs. The cost per case was $840 for single-use scopes and $1,499 for URF-V3. The URF-V3 scope exhibited a median durability of 13.5 cases before requiring repair. A cost-comparable break-even point for URF-V3 over single-use scopes was achieved in 156 cases. The single-use group required higher total laser energy use (5.0 vs. 3.0 kJ), more frequent URS-assisted access (50.9% vs. 30.3%), and longer operative times (107 vs. 95 min), with no difference in stone-free rates between the groups. Multivariate analysis revealed that scope damage was associated with supine positioning (odds ratio [OR], 6.38), pre-stenting (OR, 7.12), and higher stone density (OR, 1.01).

**Conclusion:**

Single-use flexible ureteroscopes offer economic advantages in ECIRS, particularly in complex case settings, as the aggressive use of flexible URS increases the scope damage risk.

## Introduction

The global prevalence of urolithiasis has increased from 1% to 13% [1]. Technological advancements have facilitated the increased adoption of endoscopic procedures, such as retrograde intrarenal surgery (RIRS), percutaneous nephrolithotomy (PCNL), and endoscopic combined intrarenal surgery (ECIRS) to treat kidney stones. ECIRS integrates PCNL with flexible retrograde ureteroscopy, enabling simultaneous anterograde and retrograde management of complex stones, thereby reducing the number of tract creations required [2]. Despite its benefits, the more aggressive use in ECIRS compared with that in RIRS settings contributes to a greater likelihood of scope damage and breakage, which requires additional costs [3]. Over the past decade, various single-use ureteroscopes have been developed to address this issue [4, 5]. A systematic review and meta-analysis within the RIRS context revealed no statistically significant differences in stone-free rates or incidence of complications. Furthermore, the single-use ureteroscope (URS) provided maneuverability and visibility comparable to those of a reusable URS. This indicates that the choice of URS may be more strongly influenced by logistical considerations, such as availability and cost, rather than on performance disparities [5, 6]. In this study, we conducted a cost-benefit analysis of a randomized cohort study comparing five brand-new, latest generation digital reusable scopes (URF-V3) with single-use scopes (WiScope^®^ and Uscope^®^) in the ECIRS context.

## Materials and methods

### Study design and flexible ureteroscopes

This was a single-center, randomized cohort study comparing two types of URS in an ECIRS setting conducted from September 2021 to June 2024 at Nagoya City University (NCU) Hospital. The study was approved by the Institutional Review Board of the NCU (approval number: 60-19-0044), and informed consent was obtained from all participants. All patient data were managed in accordance with the Declaration of Helsinki.

### Participants

The study population comprised patients between 20 and 80 years of age, presenting with renal and/or ureteral stones exceeding 15 mm in diameter, necessitating ECIRS. Exclusion criteria were pregnancy, uncontrolled diabetes, solitary kidneys, urinary anomalies, or urethral strictures.

Patient data including demographics, stone characteristics, grade of preoperative hydronephrosis, presence of ureteral stents and nephrostomy tubes, positive urine culture, and bacteriuria, were documented. Intraoperative data encompassed patient positioning, PNL tract size, ureteral access sheath size, ureteroscopy-assisted puncture, number of punctures, surgical procedure, fluoroscopy, laser duration, and total laser energy. Perioperative and postoperative outcomes included complications, visual stone-free rate at the end of surgery, bacteriuria, infection, stone-free status, and stone composition. Stone volume was calculated using the following formula: width × length × height. Stone-free status was categorized into grades based on maximum length: absolute stone-free (Grade A), remnants < 2 mm (Grade B), remnants 2.1–4.0 mm (Grade C), or remnants > 4.0 mm (Grade D), determined by CT scan at 3 months postoperatively.

### Surgical technique

All ECIRS procedures were performed by at least two urologists. Patients under general anesthesia were positioned in either the modified Galdakao–Valdivia or prone split-leg position at the surgeon’s discretion. Flexible cystoscopy was conducted to identify the ureteral orifice. A 0.035-mm guidewire was advanced into the renal pelvis, followed by the insertion of a ureteral access sheath (10/12, 11/13, or 12/14-French [Fr]) over the guidewire to the stone site. A flexible URS was used, with an automated irrigation system set to a pressure of 90 mmHg. Artificial hydronephrosis was induced via retrograde injection of contrast medium. Renal puncture was performed using an ultrasound-guided technique and a nephrostomy tract was created and monitored under direct vision from the flexible URS, if feasible. Stone fragmentation was achieved using LithoClast lithotripsy^®^ (Electro Medical System S.A., Nyon, Switzerland) or Ho: YAG laser through a 12-Fr mini-nephroscope (Karl Storz, Tuttlingen, Germany) and flexible URS using the Ho: YAG laser. Stone fragments were extracted from the renal puncture sites. Finally, a 6-Fr double-J ureteric stent was placed upon completion of the procedure. A nephrostomy tube was placed in cases with active bleeding. The surgical duration was recorded from the time of retrograde insertion of the flexible URS to completion of the ureteric stent and/or nephrostomy tube placement.

### Study endpoints

Five novel URF-V3 scopes were utilized in the study. Patients were randomized to either the URF-V3 or 7.5Fr flexible single-use URS group, using a computer-based algorithm sequence (UMIN Clinical Trials Registry; https://www.umin.ac.jp/ctr/, registration number: UMIN000055469) stratified according to age, sex, laterality, total stone burden, and the presence of lower calyceal stones. The study concluded that each URF-V3 requires at least one repair owing to breakage.

The primary endpoints were cost per procedure and scope durability, while the secondary endpoints were surgical outcomes, including procedural time, stone-free status, and perioperative complications.

Calculation formula.

To estimate the cost based on the number of reusable URS cases required to achieve a break-even point with the cost of the single-use URS group, we used the following formula:

$$ \begin{aligned} {\text{Cost reusable }}(N) = ({\text{Capital Cost all scopes }} + {\text{ C processing }}x{\text{ }}N{\text{ }} + {\text{ Repair Cost per case}}*)/N \\ \end{aligned} $$

^*^Repair Cost per case = (number of repairs/total cases of all reusable scopes) × cost per repair × N.

We assumed that the scope handled fewer cases after each repair, decreasing by 10% per repair.

### Statistical analysis

Continuous, normally distributed variables are expressed as means with standard deviations, whereas non-normally distributed variables are described using medians with interquartile ranges and compared using the Mann–Whitney U test or *t*-test when appropriate. Categorical variables are presented as proportions and compared using the chi-square or Fisher’s exact test. A binary logistic regression model for multivariate analysis was employed to identify factors affecting ureteroscopic damage after ECIRS. Statistical significance was set at *p* < 0.05. All statistical analyses were conducted using EZR (Saitama Medical Center, Jichi Medical University, Saitama, Japan), which serves as the graphical user interface for R (R Foundation for Statistical Computing, Vienna, Austria) [7].

## Results

After randomization, 89 patients were assigned to each URS group. No significant differences were observed between the two groups regarding age, sex, performance status, or body mass index. The single-use cohort exhibited significantly higher preoperative positive urine culture, ureteroscopic-assisted puncture, and longer surgical duration; however, the other pre-, peri-, and postoperative outcomes were comparable to those in the reusable cohort. Three types of damage were observed in the single-use group, whereas eight types were observed in the reusable group (Table 1). These included two minor damages for which the scope continued to be used until the completion of the procedures in both cohorts. Additionally, in the single-use group, one case of severe damage necessitated a scope change to a reusable scope, and six cases of severe damage required repair. At the study endpoint, one reusable scope required two repairs, whereas the others were repaired once. Table 2 presents a comparison between groups with and without scope damage. The demographics and pre-, peri-, and postoperative characteristics were comparable between the two cohorts. However, the ratio of individuals with a preoperative stent, longer surgical duration, and total laser duration was significantly higher in the cohort with damaged scopes. Multivariate analysis indicated that greater stone density, presence of a preoperative ureteral stent, and Galdakao-modified Valdivia position were significantly associated with ureteroscopic damage (Table 3).

**Table 1:** Patient demographics, stone characteristics, and comparisons of pre-, peri-, and postoperative outcomes between flexible single-use and reusable ureteroscopes

		Overall	Single-use	Reusable	*P*-value
		*N* = 178	*N* = 89	*N* = 89	
Age, years		59.26 (13.77)	59.35 (13.95)	59.17 (13.66)	0.931
Male sex, n (%)		113 (63.5)	54 ( 60.7)	59 ( 66.3)	0.534
Performance status (%)	0	160 (89.9)	76 ( 85.4)	84 ( 94.4)	0.1
	1	1 ( 0.6)	1 ( 1.1)	0 ( 0.0)	
	2	7 ( 3.9)	6 ( 6.7)	1 ( 1.1)	
	4	10 ( 5.6)	6 ( 6.7)	4 ( 4.5)	
Body mass index, kg/m^2^		24.37 (4.93)	24.74 (4.58)	24.00 (5.26)	0.314
Stone laterality, n (%)	Right	92 (51.7)	46 ( 51.7)	46 ( 51.7)	1
	Left	86 (48.3)	43 ( 48.3)	43 ( 48.3)	
Stone location, n (%)	R1	5 ( 2.8)	3 ( 3.4)	2 ( 2.2)	0.708
	R2 lower	56 (31.5)	28 ( 31.5)	28 ( 31.5)	
	R2 middle	38 (21.3)	22 ( 24.7)	16 ( 18.0)	
	R2 upper	27 (15.2)	12 ( 13.5)	15 ( 16.9)	
	R3	33 (18.5)	15 ( 16.9)	18 ( 20.2)	
	U1	17 ( 9.6)	7 ( 7.9)	10 ( 11.2)	
	U2	2 ( 1.1)	2 ( 2.2)	0 ( 0.0)	
Stone density		1398.5 [1150.5, 1562.2]	1377.0 [1100.0, 1555.0]	1406.0 [1200.0, 1565.0]	0.512
Stone volume		3594.0 [1953.0, 8842.5]	4200.0 [1950.0, 12012.0]	3150.0 [1962.0, 8316.0]	0.265
Stone size		26.0 [19.0, 35.0]	28.0 [19.0, 38.0]	25.0 [19.0, 32.0]	0.14
Staghorn, n (%)	0	122 (68.5)	57 ( 64.0)	65 ( 73.0)	0.125
	1	46 (25.8)	24 ( 27.0)	22 ( 24.7)	
	2	10 ( 5.6)	8 ( 9.0)	2 ( 2.2)	
Hydronephrosis grade, n (%)	0	105 (59.0)	58 ( 65.2)	47 ( 52.8)	0.441
	1	23 (12.9)	11 ( 12.4)	12 ( 13.5)	
	2	23 (12.9)	10 ( 11.2)	13 ( 14.6)	
	3	23 (12.9)	8 ( 9.0)	15 ( 16.9)	
	4	4 ( 2.2)	2 ( 2.2)	2 ( 2.2)	
Preoperative ureteral stent, n (%)		35 (19.7)	22 ( 24.7)	13 ( 14.6)	0.131
Preoperative percutaneous nephrostomy, n (%)		16 ( 9.0)	9 ( 10.1)	7 ( 7.9)	0.794
Preoperative urine culture, n (%)	Positive	56 (31.5)	37 ( 41.6)	19 ( 21.3)	0.006
Preoperative bacteriuria, n (%)	Positive	53 (30.6)	31 ( 35.2)	22 ( 25.9)	0.422
Position, n (%)	Prone	116 (65.2)	56 ( 62.9)	60 ( 67.4)	0.637
	Valdivia	62 (34.8)	33 ( 37.1)	29 ( 32.6)	
Tract size (%)	12 Fr	1 ( 0.6)	1 ( 1.1)	0 ( 0.0)	0.247
	16 Fr	2 ( 1.1)	0 ( 0.0)	2 ( 2.2)	
	17.5 Fr	170 (96.0)	85 ( 96.6)	85 ( 95.5)	
	19 Fr	1 ( 0.6)	0 ( 0.0)	1 ( 1.1)	
	21 Fr	2 ( 1.1)	2 ( 2.3)	0 ( 0.0)	
	22 Fr	1 ( 0.6)	0 ( 0.0)	1 ( 1.1)	
Ureteral access sheath (%)	10/12Fr	126 (79.2)	61 ( 77.2)	65 ( 81.2)	0.697
	11/13Fr	7 ( 4.4)	3 ( 3.8)	4 ( 5.0)	
	12/14Fr	26 (16.4)	15 ( 19.0)	11 ( 13.8)	
Ureteroscopic assisted puncture (%)		72 (40.4)	45 ( 50.6)	27 ( 30.3)	0.009
Number of punctures		2.00 [1.00, 3.00]	2.00 [1.00, 3.00]	2.00 [1.00, 3.00]	0.341
Surgical duration, min		95.00 [82.00, 119.75]	107.00 [83.00, 135.00]	94.00 [78.00, 111.00]	0.03
Fluoroscopic duration, s		486.00 [300.00, 723.00]	573.00 [300.00, 740.00]	445.00 [300.00, 720.00]	0.262
Total laser duration, s		380.00 [98.00, 757.00]	450.00 [117.50, 825.50]	317.00 [102.00, 665.25]	0.189
Total laser energy, kJ		9.33 (19.53)	12.47 (25.08)	6.66 (12.65)	0.06
Perioperative complication grade, n (%)	grade 0	162 (92.0)	80 ( 90.9)	82 ( 93.2)	0.92
	grade 1	3 ( 1.7)	1 ( 1.1)	2 ( 2.3)	
	grade 2	8 ( 4.5)	4 ( 4.5)	4 ( 4.5)	
	grade 3	1 ( 0.6)	1 ( 1.1)	0 ( 0.0)	
	grade 4	1 ( 0.6)	1 ( 1.1)	0 ( 0.0)	
	NA	1 ( 0.6)	1 ( 1.1)	0 ( 0.0)	
Intraoperative scope damage, n (%)		11 ( 6.2)	3 ( 3.4)	8 ( 9.0)	0.212
Postop bacteriuria, n (%)		41 (23.0)	24 (28.6)	17 (19.1)	0.143
Postop infection, n (%)		8 ( 4.6)	4 ( 4.6)	4 ( 4.5)	1
Visual stone clearance, n (%)		157 (88.2)	75 ( 84.3)	82 ( 92.1)	0.162
Stone free status at 3 months after surgery, n (%)	Grade A	79 (44.6)	37 ( 41.6)	42 ( 47.7)	0.72
	Grade B	23 (13.0)	13 ( 14.6)	10 ( 11.4)	
	Grade C	17 ( 9.6)	7 ( 7.9)	10 ( 11.4)	
	Grade D	38 (21.5)	20 ( 22.5)	18 ( 20.5)	
	NA	20 (11.3)	12 ( 13.5)	8 ( 9.1)	
Stone composition (%)	CaOX	124 (73.4)	53 ( 64.6)	71 ( 81.6)	0.157
	CaP	16 ( 9.5)	10 ( 12.2)	6 ( 6.9)	
	UA	4 ( 2.4)	3 ( 3.7)	1 ( 1.1)	
	Cystine	4 ( 2.4)	3 ( 3.7)	1 ( 1.1)	
	MAP	19 (11.2)	11 ( 13.4)	8 ( 9.2)	
	Others	2 ( 1.2)	2 ( 2.4)	0 ( 0.0)	

**Table 2:** Patient demographics, stone characteristics, and comparisons of pre-, peri-, and postoperative outcomes between groups with and without ureteroscope damage

		Non-damage	Damage	*P*- value
		*N* = 167	*N* = 11	
Age, years		59.20 (13.81)	60.09 (13.84)	0.837
Male sex, n (%)		105 ( 62.9)	8 ( 72.7)	0.748
Performance status (%)	0	149 ( 89.2)	11 (100.0)	1
	1	1 ( 0.6)	0 ( 0.0)	
	2	7 ( 4.2)	0 ( 0.0)	
	4	10 ( 6.0)	0 ( 0.0)	
Body mass index, kg/m^2^		24.29 (4.78)	25.61 (7.01)	0.39
Stone laterality, n (%)	Right	87 ( 52.1)	5 ( 45.5)	0.761
	Left	80 ( 47.9)	6 ( 54.5)	
Stone location, n (%)	R1	5 ( 3.0)	0 ( 0.0)	0.842
	R2 lower	53 ( 31.7)	3 ( 27.3)	
	R2 middle	35 ( 21.0)	3 ( 27.3)	
	R2 upper	24 ( 14.4)	3 ( 27.3)	
	R3	32 ( 19.2)	1 ( 9.1)	
	U1	16 ( 9.6)	1 ( 9.1)	
	U2	2 ( 1.2)	0 ( 0.0)	
Stone density		1377.0 [1134.5, 1557.5]	1474.0 [1312.0, 1720.5]	0.095
Stone volume		3588.0 [1956.0, 8736.0]	6210.0 [1500.0, 13334.0]	0.894
Stone size		26.0 [19.0, 35.0]	28.0 [24.5, 40.5]	0.453
Staghorn (%)	0	117 ( 70.1)	5 ( 45.5)	0.104
	1	40 ( 24.0)	6 ( 54.5)	
	2	10 ( 6.0)	0 ( 0.0)	
Hydronephrosis grade (%)	0	100 ( 59.9)	5 ( 45.5)	0.56
	1	20 ( 12.0)	3 ( 27.3)	
	2	22 ( 13.2)	1 ( 9.1)	
	3	21 ( 12.6)	2 ( 18.2)	
	4	4 ( 2.4)	0 ( 0.0)	
Preoperative ureteral stent, n (%)		30 ( 18.0)	5 ( 45.5)	0.042
Preoperative percutaneous nephrostomy, n (%)		14 ( 8.4)	2 ( 18.2)	0.258
Urine culture, n (%)	positive	52 ( 31.1)	4 ( 36.4)	0.743
Preoperative bacteriuria, n (%)	positive	47 ( 29.0)	6 ( 54.5)	0.168
Position, n (%)	Prone	112 ( 67.1)	4 ( 36.4)	0.051
	Valdivia	55 ( 32.9)	7 ( 63.6)	
Tract size (%)	12 Fr	1 ( 0.6)	0 ( 0.0)	1
	16 Fr	2 ( 1.2)	0 ( 0.0)	
	17.5 Fr	159 ( 95.8)	11 (100.0)	
	19 Fr	1 ( 0.6)	0 ( 0.0)	
	21 Fr	2 ( 1.2)	0 ( 0.0)	
	22 Fr	1 ( 0.6)	0 ( 0.0)	
Urereral access sheath (%)	10/12Fr	117 ( 79.1)	9 ( 81.8)	0.528
	11/13Fr	6 ( 4.1)	1 ( 9.1)	
	12/14Fr	25 ( 16.9)	1 ( 9.1)	
Ureteroscopic assisted puncture, n (%)		67 ( 40.1)	5 ( 45.5)	0.759
Number of punctures		2.00 [1.00, 3.00]	2.00 [1.00, 3.50]	0.798
Surgical duration, min		95.00 [80.50, 117.50]	120.00 [103.00, 155.50]	0.025
Fluoroscopic duration, s		484.50 [300.00, 720.00]	528.00 [313.00, 976.50]	0.517
Total laser duration, s		366.00 [95.75, 707.50]	1003.00 [393.00, 1844.50]	0.027
Total laser energy, kJ		9.00 (19.93)	13.85 (12.60)	0.429
Perioperative complication grade. n (%)	grade 0	153 ( 92.2)	9 ( 90.0)	0.35
	grade 1	2 ( 1.2)	1 ( 10.0)	
	grade 2	8 ( 4.8)	0 ( 0.0)	
	grade 3	1 ( 0.6)	0 ( 0.0)	
	grade 4	1 ( 0.6)	0 ( 0.0)	
	NA	1 ( 0.6)	0 ( 0.0)	
Scope type, n (%)	0	86 ( 51.5)	3 ( 27.3)	0.212
	1	81 ( 48.5)	8 ( 72.7)	
Postop bacteriuria, n (%)		40 (24.7)	1 ( 9.1)	0.53
Postop infection, n (%)		8 ( 4.8)	0 ( 0.0)	1
Visual stone clearance, n (%)		148 ( 88.6)	9 ( 81.8)	0.622
Stone free status at 3 months after surgery, n (%)	Grade A	76 ( 45.5)	3 ( 30.0)	0.629
	Grade B	21 ( 12.6)	2 ( 20.0)	
	Grade C	16 ( 9.6)	1 ( 10.0)	
	Grade D	36 ( 21.6)	2 ( 20.0)	
	NA	18 ( 10.8)	2 ( 20.0)	
Stone composition (%)	CaOX	114 ( 71.7)	10 (100.0)	0.65
	CaP	16 ( 10.1)	0 ( 0.0)	
	UA	4 ( 2.5)	0 ( 0.0)	
	Cystine	19 ( 11.9)	0 ( 0.0)	
	MAP	2 ( 1.3)	0 ( 0.0)	
	Others	4 ( 2.5)	0 ( 0.0)	

**Table 3:** Multivariate analysis for scope damage

Factor	Odds ratio (95% CI*)	*P*-value
Age	0.98 (0.93–1.03)	0.43
Male sex	1.96 (0.36–10.70)	0.44
Body mass index	1.13 (0.96–1.32)	0.13
Stone density	1.00 (1.00–1.01)	0.044
Stone volume	1.00 (1.00–1.00)	0.22
Lower caliceal stone	1.85 (0.29–11.90)	0.52
Preoperative ureteral stent	7.12 (1.42–35.80)	0.017
Galdakao-modified Valdivia position	6.38 (1.09–37.40)	0.04
Ureteral access sheath 12/14Fr	0.51 (0.04–6.62)	0.61
Surgical duration	1.02 (0.99–1.04)	0.18
Total laser duration	1.00 (1.00–1.00)	0.31

* CI; Confidence interval

The cost per case was $840 for single-use scopes and $1,516 for reusable scopes, considering a median repair cost of $2,815 per scope and a processing cost of $28 per procedure using reusable scopes. The reusable scope exhibited a median durability of 13.5 cases before requiring initial repair. Based on our cost assumption formula for a reusable URS, the cost-equivalent breakeven point for URF-V3 over single-use scopes was anticipated to be achieved at 156 cases (Table 4).

**Table 4:** Total cost per case between flexible single-use and reusable ureteroscopes

	Single-use	Reusable
	*N* = 89	*N* = 89
Capital /case	$840	$1298
Reprocessing /case (washing, drying, and sterilization)		$28
Repair cost / per repair		$2,815
Total cost per case	$840	$1,515

## Discussion

ECIRS was introduced to achieve a higher stone-free status for large kidney and ureteral stones with fewer tracts and procedures than PCNL. However, the durability of the scope and the damage attributed to its aggressive use for complicated stones, along with the additional repair costs, are significant concerns in practice. The present study revealed that single-use scopes offer cost advantages over reusable scopes in the context of ECIRS.

Despite the high initial purchase price of reusable ureteroscopes, repeated use could theoretically make them cost-effective over time. However, considering the necessity of maintenance, sterilization, processing, and repair of damage, cumulative costs can become substantial, particularly in light of potential device malfunctions. Several single-use ureteroscopes have been developed to address these limitations. Due to differences in environments, settings, processing labor, and other factors, several studies have reported that single-use ureteroscopes have comparable or lower overall costs per procedure when factoring in significant maintenance and repair expenses, whereas others indicate that reusable scopes offer greater cost-effectiveness [8–12]. To our knowledge, our study is the first to compare the cost-effectiveness of single-use ureteroscopes in an ECIRS setting, utilizing five new reusable digital ureteroscopes. This unique analysis could provide insights into the number of cases required to achieve a cost-comparable break-even point for reusable scopes over single-use scopes in real-world settings. A systematic review and meta-analysis indicated that the calculated weighted repair rate was 6.5%, equivalent to 15 ureteroscopy procedures before repair, at a cost of 441 USD per reusable procedure [13]. Consistent with this, our study found that the median number of cases before digital reusable scopes required repair was 13.5. A predicted cost-comparable break-even point for digital reusable scopes over single-use scopes was reached at 156 cases, which aligns with a report by Yazici et al.. A reusable flexible URS can be utilized for approximately 87–133 cases, with a median of 107 cases requiring replacement in the RIRS setting [14]. This estimate is influenced by various factors, including the frequency and complexity of procedures, methods of sterilization, handling techniques, and maintenance practices, suggesting that its use in an ECIRS setting might lead to early total breakage.

Ureteroscopic damage occurred in 11 cases: three involving single-use scopes and eight in reusable scopes. Even after the initial use of a single-use URS, ECIRS resulted in some degree of scope damage, suggesting a higher likelihood that the accumulation of reusable scope damage could lead to complete device malfunction. Multivariate analysis further revealed that greater stone density, presence of a preoperative ureteral stent, and Galdakao-modified Valdivia position were factors that significantly increased the risk of ureteroscopic damage. Higher stone density necessitates longer surgical and laser times, which are associated with a greater likelihood of scope damage. Univariate analysis further demonstrated that the patients with scope damage had longer surgical and laser durations. The reasons for the increased scope damage stemming from preoperative ureteral stent placement and Valdivia position remain unclear; however, we postulate that cases requiring a preoperative stent may involve obstructive and complex stones, potentially leading to scope damage. Additionally, the subanalysis indicated that cases employing the Valdivia position had longer laser durations (314 s vs. 475 s, *p* = 0.027), contributing to scope damage.

Univariate analysis indicated that the single-use group required higher total laser energy use, more frequent URS-assisted access, and longer operative times. We assumed that the reduced concern about scope damage compared with reusable scopes may allow for more aggressive use of scopes [15], including URS-assisted access for challenging anatomical scenarios or complex stone, associated with prolonged lithotripsy, which led to a higher total laser energy and longer operative time. Although we did not assess the surgeon’s mindset when using each scope, questionnaires for clinicians may be helpful for this purpose.

Several systematic reviews and a pooled analysis have shown no significant differences in stone-free rates or postoperative complications, suggesting that both types are equally effective and safe for stone removal procedures [15, 16]. A separate meta-analysis reported a significantly higher stone-free rate for single-use ureteroscopes compared with reusable ones, while another review indicated a safety advantage for single-use ureteroscopes, associating them with lower occurrences of specific Clavien–Dindo grade II postoperative complications compared with reusable ureteroscopes [17, 18]. In line with these findings, our previous report demonstrated that a single-use URS was associated with a reduced risk of urinary tract infection and an improved stone clearance rate following ureteroscopy for urolithiasis [19]. In this study, no differences were identified concerning peri- and postoperative outcomes. Since the study was completed after all scopes had at least one breakage, these reusable scopes could still be used much more often, suggesting that prolonged use of the scopes might lead to inferior outcomes in the reusable scope cohort.

Regarding the environmental footprint of single-use versus reusable scopes, a recent systematic review indicated comparable carbon footprints for both ureteroscopes [20]. Given that CO_2_ emissions can be diminished by implementing resource efficiency strategies to enhance the preparation of reusable equipment and improve recycling protocols for single-use devices [21], surgeons should take these factors into consideration.

We must acknowledge that the current study has limitations. First, our research was conducted at an academic center that provided training for less experienced doctors and involved more complicated cases, which may not be representative of other types of facilities. In these settings, the risk of catastrophic damage before the break-even point could be real. Institutions must therefore individualize their strategy based on local procedural volume and case mix.

Second, we did not consider additional micro-cost factors such as labor and long-term performance, as previously reported [8]. Third, there was a potential observational bias in this prospective study that allowed for the management of procedures during the study period, which may have affected the results. Despite these limitations, our findings regarding the cost and frequency of damage to the URS are novel and may provide insights into purchase decisions and clinical indications for the use of reusable versus single-use ureteroscopes in ECIRS.

In conclusion, single-use flexible ureteroscopes offer economic advantages in ECIRS, particularly in complex case settings, as the aggressive use of flexible URS increases the risk of scope damage.

## Data Availability

The authors have agreed to data sharing if readers wish to assess.
